# Increased Motor-Impairing Effects of the Neuroactive Steroid Pregnanolone in Mice with Targeted Inactivation of the GABA_A_ Receptor γ2 Subunit in the Cerebellum

**DOI:** 10.3389/fphar.2016.00403

**Published:** 2016-10-27

**Authors:** Elli Leppä, Anni-Maija Linden, Maria I. Aller, Peer Wulff, Olga Vekovischeva, Bernhard Luscher, Hartmut Lüddens, William Wisden, Esa R. Korpi

**Affiliations:** ^1^Department of Pharmacology, Faculty of Medicine, University of HelsinkiHelsinki, Finland; ^2^Instituto de Neurociencias, Consejo Superior de Investigaciones Científicas, Universidad Miguel Hernández de ElcheSan Juan de Alicante, Spain; ^3^Institute of Physiology, University of KielKiel, Germany; ^4^Department of Biology, The Pennsylvania State UniversityUniversity Park, PA, USA; ^5^Department of Psychiatry and Psychotherapy, University Medical Center of the Johannes Gutenberg-University MainzMainz, Germany; ^6^Department of Life Sciences, Imperial College LondonLondon, UK

**Keywords:** extrasynaptic GABA_A_ receptors, neurosteroids, cerebellum, motor performance, Purkinje cells

## Abstract

Endogenous neurosteroids and neuroactive steroids have potent and widespread actions on the brain via inhibitory GABA_A_ receptors. In recombinant receptors and genetic mouse models their actions depend on the α, β, and δ subunits of the receptor, especially on those that form extrasynaptic GABA_A_ receptors responsible for non-synaptic (tonic) inhibition, but they also act on synaptically enriched γ2 subunit-containing receptors and even on αβ binary receptors. Here we tested whether behavioral sensitivity to the neuroactive steroid agonist 5β-pregnan-3α-ol-20-one is altered in genetically engineered mouse models that have deficient GABA_A_ receptor-mediated synaptic inhibition in selected neuronal populations. Mouse lines with the GABA_A_ receptor γ2 subunit gene selectively deleted either in parvalbumin-containing cells (including cerebellar Purkinje cells), cerebellar granule cells, or just in cerebellar Purkinje cells were trained on the accelerated rotating rod and then tested for motor impairment after cumulative intraperitoneal dosing of 5β-pregnan-3α-ol-20-one. Motor-impairing effects of 5β-pregnan-3α-ol-20-one were strongly increased in all three mouse models in which γ2 subunit-dependent synaptic GABA_A_ responses in cerebellar neurons were genetically abolished. Furthermore, rescue of postsynaptic GABA_A_ receptors in Purkinje cells normalized the effect of the steroid. Anxiolytic/explorative effects of the steroid in elevated plus maze and light:dark exploration tests in mice with Purkinje cell γ2 subunit inactivation were similar to those in control mice. The results suggest that, when the deletion of γ2 subunit has removed synaptic GABA_A_ receptors from the specific cerebellar neuronal populations, the effects of neuroactive steroids solely on extrasynaptic αβ or αβδ receptors lead to enhanced changes in the cerebellum-generated behavior.

## Introduction

Endogenous neurosteroids and synthetic neuroactive steroids are among the most potent and efficacious modulators of the brain’s main ligand-gated inhibitory neurotransmitter receptor, the γ-aminobutyric acid type A (GABA_A_) receptor, producing wide-ranging effects on behavior, including motor relaxation and impairment (Baulieu et al., 2001; Belelli et al., 2006). The concentrations of neurosteroids in the brain are increased in stress and by drugs, such as alcohol and antidepressants (Barbaccia, 2004). Endogenous concentrations of neurosteroids fluctuate during the ovarian cycle and in pregnancy, inducing changes in GABA_A_ receptor subtype populations, particularly in those containing the δ subunits (Maguire et al., 2005; Uusi-Oukari and Korpi, 2010; Wu et al., 2013; Mackenzie and Maguire, 2014), which may result in impaired regulation of mood in some patients (Backstrom et al., 2011, 2014). Neurosteroid mechanisms have been implicated in acute liver failure and hepatic encephalopathy (Ahboucha et al., 2012). Furthermore, allopregnanolone, a metabolite of progesterone and one of the main endogenous GABAergic neurosteroids, promotes proliferation of neural progenitor cells *in vitro* via activation of GABA_A_ receptors and, after a single dose, enhances neurogenesis in the hippocampal subgranular zone and in the dopaminergic substantia nigra *pars compacta* of adult transgenic model mice for Alzheimer’s disease (Wang et al., 2010; Zhang et al., 2015). However, chronic treatment with allopregnanolone accelerates Alzheimer’s disease in several types of mouse models (Bengtsson et al., 2012, 2013). There appears to be a need to better understand the mechanisms underlying neurosteroid sensitivity.

The mechanisms of neurosteroid sensitivity remain unclear. Neurosteroid actions can be mediated by all subtypes of GABA_A_ receptors, with just α and β subunits giving full potency and efficacy (Hosie et al., 2006). The type of the α subunit mediates some of the variation noted in receptor properties, whereas the type of the β subunit has less influence. In recombinant receptors, inclusion of a γ2 subunit has little effect on binding or efficacy compared to binary αβ receptors, but the inclusion of the δ subunit strongly increases the GABA-potentiating effects of neurosteroid agonists (Belelli et al., 2002; Wohlfarth et al., 2002). Neurosteroids affect both synaptic (mainly γ2-GABA_A_ dependent) and extrasynaptic (mainly δ-GABA_A_ dependent) inhibition in brain slices, but, in line with the increased efficacy of neurosteroids on δ subunit-containing receptors, their behavioral and neurophysiological effects are strongly reduced when extrasynaptic GABAergic inhibition has been attenuated, e.g., in the GABA_A_ receptor δ subunit knockout mice (Mihalek et al., 1999; Spigelman et al., 2003; Stell et al., 2003). In the case of extrasynaptic GABA_A_ receptors containing the α4 and δ subunits, these mechanisms involve protein kinase C activation, and subsequent GABA_A_ α4 subunit phosphorylation, increased membrane insertion of these receptors, leading to enhanced tonic conductance by prolonged exposure to neurosteroids (Abramian et al., 2014). Thus, whereas neurosteroid agonists affect both synaptic and extrasynaptic receptors, their behavioral effects seem to primarily depend on extrasynaptic receptors. It is evident that more information is needed on the effects of GABA_A_ receptor subunit composition on neurosteroid sensitivity at the whole animal level.

In the present experiments, we genetically removed the γ2 subunit in discrete neuronal populations. We used male mice to avoid estrus cycle-associated neurosteroid fluctuations. Using a simple cerebellum-related behavioral task on rotating rods, the mouse models enabled us to ascertain how the γ2 subunit affects the sensitivity of the neuroactive steroid pregnanolone in cerebellar circuitry. The cerebellum was very suitable for these experiments, since the two main targets we used (Purkinje and granule cells) are known to express the γ2 subunit and a limited number of other subunits [Purkinje cells α1, β2/3 and γ2; granule cells α1/6, β2/3, δ and γ2 (Wisden et al., 1996)]. The contributions of extrasynaptic and synaptic GABA_A_ receptors in mediating pregnanolone effects were further validated by transgenic re-introduction of the γ2 subunit into Purkinje cells (PC-γ2-swap mice) (Wulff et al., 2007; Wisden et al., 2009).

## Materials and Methods

### Animals

All mice used for experiments were male. Parvalbumin (Pv)-neuron GABA_A_ γ2 subunit knockout mice (Pv-Δγ2) and littermate control Pv-Cre mice were generated as described (Wulff et al., 2009a), by crossing Pv-Cre mice with γ2I77 lox mice (Fuchs et al., 2007; Wulff et al., 2007). Pv-Δγ2 mice were important for the study, since cerebellar Purkinje cells and molecular layer interneurons are positive for parvalbumin (Leppa et al., 2011), and thus lose synaptic inhibition. Cerebellar granule cell (Gr) GABA_A_ γ2 subunit knockout (Gr-Δγ2) homozygous (-/-), heterozygous (+/-) and wild-type (+/+) littermate mice were generated by crossing α6-Cre mice [B6.129P2-*Gabra6^tm2(cre)Wwis^*/Mmucd mouse mutant resource stock MMRRC:015968-UCD (Aller et al., 2003)] with a mouse line containing a γ2 gene flanked by lox P sites [Jax lab STOCK No. 016830, *Gabrg2*^tm2Lusc^/J, also known as fγ2 mice (Schweizer et al., 2003)]. Gr-Δγ2 mice were used to study the effect of γ2 subunit deficiency in only one major cerebellar neuron population. Cerebellar Purkinje cell (PC) GABA_A_ γ2 subunit knockout mice (PC-Δγ2) and littermate control γ2I77lox mice were generated as described (Wulff et al., 2007), by crossing γ2I77lox mice with L7-Cre mice (Barski et al., 2000; Wulff et al., 2007). The γ2I77lox mice are available at JAX labs, stock STOCK 021197 *Gabrg2*^tm1Wul^/J, and have loxP sites surrounding exon 4 of the γ2 subunit gene (Wulff et al., 2007). The γ2I77lox mice have the GABA_A_ receptor γ2 subunit F77 residue point-mutated to encode I77, causing an inability of the γ2 subunit-dependent benzodiazepine binding site to mediate pharmacological effects of the sedative-hypnotic zolpidem and the β-carboline convulsant 3-carbomethoxy-4-ethyl-6,7-dimethoxy-β-carboline (Cope et al., 2005; Leppa et al., 2005) and to bind with high affinity the universal benzodiazepine site ligand [^3^H]Ro 15-4513 (Leppa et al., 2011; Linden et al., 2011) (see **Figure 3C**). In other aspects the γ2I77 subunit-containing GABA_A_ receptors function normally (Cope et al., 2005). PC GABA_A_ γ2 subunit swap mice (PC-γ2-swap) were generated by expressing the wildtype γ2 subunit under the control of Purkinje-cell specific L7-promoter to restore wildtype γ2 expression in PCs following the specific inactivation of γ2 I77 subunit in these cells (in PC-Δγ2 mice). The PC-γ2-swap mice have been previously used to selectively restore zolpidem sensitivity in Purkinje cells to study how selective inhibition of PCs by zolpidem affects motor performance (Wulff et al., 2007).

For all the conditional mouse crosses, all experiments were performed on mice homozygous for the conditional γ2 allele and hemizygous for the Cre transgenes; Cre-negative mice served as the littermate controls. The mice were 4–10 months old when used. Weights at the rotarod testing: Pv-Δγ2 mice and their Pv-Cre/γ2I77 controls 22 ± 3 (*n* = 7) and 25 ± 2 (8) g (mean ± SD), respectively; Gr-Δγ2+/+, +/-, and -/- mice 32 ± 4 (5), 31 ± 2 (10), and 29 ± 1 (6) g, respectively; PC-Δγ2, γ2I77lox and PC-γ2-swap mice 25 ± 4 (5), 25 ± 6 (10) and 24 ± 2 (6) g, respectively. The animals were housed (1–5 per cage) in transparent polypropylene Makrolon cages with standard rodent pellets (Harlan Teklad Global Diet, Bicester, UK) and tap water *ad lib*. Lights were on from 7 a.m. to 7 p.m.

All behavioral animal experiments were carried out with the permissions (ESLH-2004-01605/Ym-23 and ESLH-2006-09005/Ym-23) of the State Provincial Government of Southern Finland, the governing body that oversaw animal ethics for the University of Helsinki. All efforts were made to minimize the number and suffering of animals. About 1 week after behavioral tests, the animals were strongly sedated with CO_2_, decapitated, and brains dissected out for further analyses.

### Motor Tests

To investigate the motor coordination capabilities of each mouse line rotarod tests were performed (Korpi et al., 1999). The mice were trained during 7 days (4–6 trials per day) to stay on a rotating rod (diameter 4 cm, Rotamex 4/8, Columbus Instruments, Columbus, OH, USA) for 180 s, with the rotation speed being linearly accelerated from 5 to 30 rpm. Due to the impairment in motor performance of Pv-Δγ2 mice, a lower speed of 5 to 20 rpm was used in the training of these and their littermate control mice. The latency to fall from the rod in each trial was recorded and a daily average of 4–6 trials was calculated for each animal.

To study the sensitivities of the mouse lines to neurosteroids a synthetic neuroactive steroid 5β-pregnan-3α-ol-20-one (pregnanolone, Sigma–Aldrich Chemical Company, St. Louis, MO, USA) was used. Well-trained animals were injected with vehicle or pregnanolone at 30 min intervals, 15 min before each single-trial rotarod testing. Drug dosing was cumulative, in order to reduce the number of tested animals (vehicle+10+10+10 mg pregnanolone/kg, for the total dose of 30 mg/kg). Cumulative dosing has been used successfully in previous dose-response studies for other drugs (Cope et al., 2004; Wulff et al., 2007). Pregnanolone was dissolved overnight in cremophor (Cremophor EL, Sigma) and brought to concentration with physiological saline (final concentration 30% cremophor, which was used without pregnanolone as a vehicle control). Pregnanolone and vehicle solutions were injected i.p. in a volume of 10 ml/kg body weight.

### Tests for Anxiolytic and Explorative Activity

To compare the PC-mouse lines in the effects of pregnanolone on the level of anxiety and explorative activity, we used a batch of mice that was naïve to behavioral tests in elevated plus-maze test and light:dark exploration test, as described in (Saarelainen et al., 2008; Leppa et al., 2011), with 1 week washout between the tests.

The elevated plus-maze test was performed on the apparatus made of gray plastic and elevated to 50 cm from the floor level. It consisted of a central platform (5 cm × 5 cm), from which two open arms (5 cm × 40 cm with a 0.7 cm ledge) and two enclosed arms (5 cm × 40 cm × 20 cm) extended. The mice were placed individually on the central platform facing an open arm and allowed free exploration of the maze for 5 min with their behavior being recorded using a video tracking system with a CCD video camera above the plus maze. This was done 15 min after the injection of vehicle or pregnanolone (10 mg/kg, i.p.), which was done in a balanced order. The position and movements of the center of the animal’s surface area were analyzed automatically using EthoVision software Color-Pro 3.0 software (Noldus Information Technology, Wageningen, Netherlands). The central area was extended to include the first 2 cm of each arm. An arm entry was recorded when the center of the mouse entered the distal part of the arm. This corresponds to the definition of an arm entry with all four legs on the arm. During 5-min testing periods the time spent on the open arms, the number of entries into the arms, and the total distance traveled in the maze were recorded and analyzed by EthoVision. The plus maze was carefully cleaned with water-moistened paper towel and dried after each mouse, thus avoiding any aversive smells to carry over to the next mouse. The mice were returned to their home cage when all mice from the same cage were tested.

The light:dark test (Saarelainen et al., 2008) was started by placing a mouse in the lit compartment of two-compartment box (47 cm × 29 cm × 35 cm) divided into one dark (16 cm × 29 cm) and one lit (31 cm × 29 cm; about 450 lux) area with open door (7 cm × 8 cm) between them. This was done 15 min after the injection of vehicle or pregnanolone. In this test, we used a slightly lower dose (7 mg/kg, i.p.) than in the elevated plus-maze test to ensure that any sensitivity differences in anxiolytic effects would be detectable. During 5-min testing periods the time spent in the lit compartment, the number of crossings between compartments and the distance traveled in the lit compartment were recorded and analyzed by EthoVision. The test-box was cleaned and dried after each mouse as was done in the elevated plus-maze test, and the mice were returned to their home cage when all mice from the same cage were tested.

### Ligand Autoradiography

Autoradiography of mouse brain horizontal 14-μm-thick cryostat sections was performed as described (Korpi and Luddens, 1997; Makela et al., 1997). For [^3^H]Ro 15-4513 autoradiography the sections were incubated at 4°C for 60 min with 15 nM [^3^H]Ro 15-4513 (Perkin-Elmer Life Sciences Inc., Waltham, MA, USA). Non-specific binding was determined with 10 μM Ro 15-1788 (flumazenil, Tocris Bioscience, MS, USA). For [^35^S]TBPS autoradiography the sections were incubated with 6 nM [^35^S]-*t*-butylbicyclophosphorothionate ([^35^S]TBPS, Perkin-Elmer) in the incubation buffer (50 mM Tris-HCl, 120 mM NaCl, pH 7.4) at room temperature for 90 min. Non-specific binding was determined with 100 μM picrotoxinin (Sigma). After the washing and drying, the sections were exposed to Kodak Biomax MR film for 1–24 weeks with ^3^H (for [^3^H]Ro 15-4513) or ^14^C (for [^35^S]TBPS) radioactivity standards (Amersham Biosciences corp., Piscataway, NJ, USA). Binding densities in the multiple locations of the granule cell and/or molecular layers of the cerebellum were quantitated with MCID M5-imaging software (Imaging Research Inc., St. Catherines Ontario, ON, Canada) and converted to radioactivity values on the basis of the simultaneously exposed standards. Non-specific binding was subtracted from all values. Importantly, in [^3^H]Ro 15-4513 autoradiography, the radioligand concentration (15 nM) was about three times higher than the dissociation constant Kd of the binding (Sieghart, 1995). Thus, the images mainly reflected the number of binding sites.

### Ligand Binding to Recombinant Receptors

Human embryonic kidney cells [HEK 293 cells; German collection of microorganisms and cell cultures (DSMZ), Braunschweig, Germany] were grown to <50% confluency on 15-cm tissue plates in 20 ml DMEM supplemented with 10% heat-inactivated fetal calf serum, 5 mM glutamine as well as penicillin and streptomycin. Transfection was carried out with a Ca^2+^-phosphate precipitation method essentially as described (Jordan et al., 1996). Briefly, plasmids were diluted in 1 ml/ plate of 0.3125 M CaCl_2_ in H_2_O. One ml/ plate of 2x HBS (274 mM NaCl; 1.5 mM Na_2_HPO_4_; 54.6 mM HEPES/NaOH; pH 7.0) was added to the DNA and incubated for 90 s. Two ml of the mixture were pipetted into 15-cm plates that were incubated for 18–24 h before the transfection medium was replaced by fresh medium. Double and triple combinations of rat GABA_A_ receptor cDNAs in eukaryotic expression vectors (Pritchett and Seeburg, 1990) of the α1, β3, γ2S, and δ subunits were employed. Final concentrations (μg vector DNA per 15 cm tissue culture plate) were: α1, 2.5; β3, 0.5; γ2S, 0.375, and δ, 2.5.

Cell membranes were prepared as described (Korpi and Luddens, 1997). Resuspended crude cell membranes (50–200 μg protein per tube) were incubated in a final volume of 0.5 ml of 50 mM Tris/citrate buffer supplemented with 0.2 M NaCl, pH 7.3, with 3 nM [^3^H]EBOB ([^3^H]ethynylbicycloorthobenzoate, NEN) with or without pregnanolone in the presence or absence of GABA. Pregnanolone was made up in DMSO at a stock of 10 mM in DMSO. GABA was diluted from a 100 mM solution in H_2_O. Binding assay procedure was performed as described earlier (Korpi and Luddens, 1997).

### Statistical Analyses

Statistical tests were performed with SPSS Software (SPSS 12.0.1, SPSS Inc., Chicago, IL, USA) or GraphPad Prism software (Prism 6.0, GraphPad Software Inc., San Diego, California, USA). Treatment groups and mouse lines were compared with either repeated measures ANOVA, one-way ANOVA or two-way ANOVA followed by Newman–Keuls *post hoc* test or Dunnett’s test. In all statistical tests the level of significance was set at *P* < 0.05.

## Results

**FIGURE 1:**
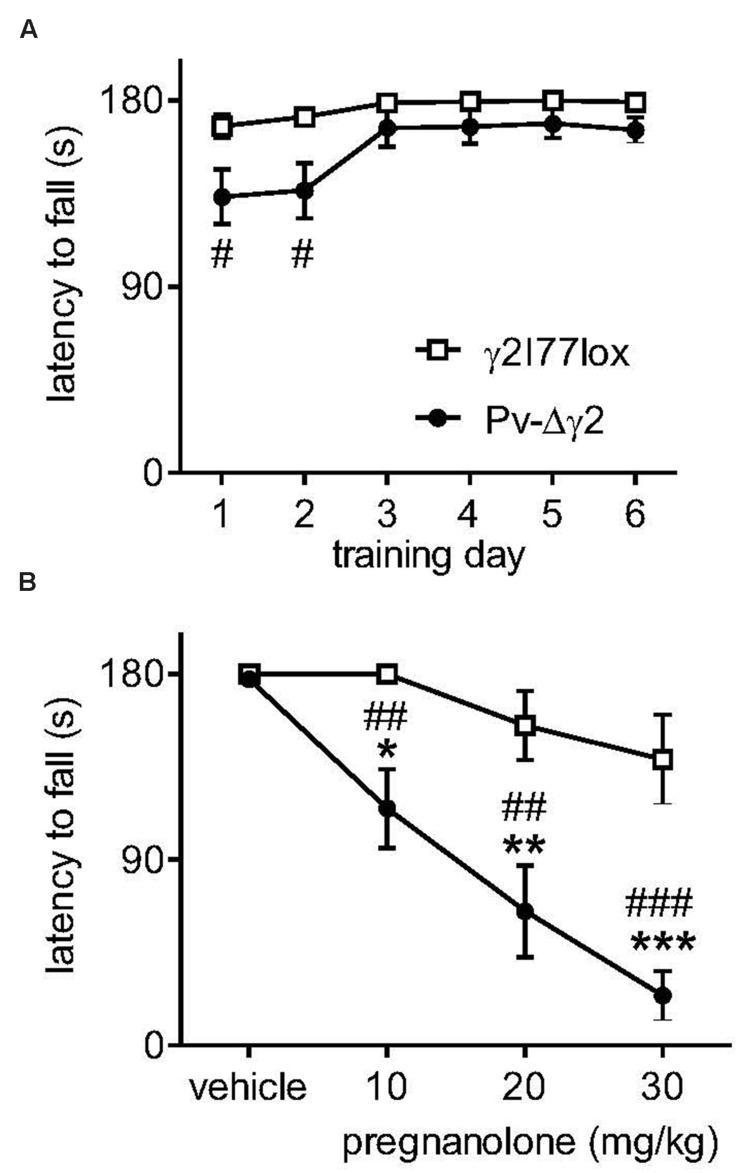
**Neurosteroid sensitivity of Pv-Δγ2 mice with targeted inactivation of GABA_A_ receptor γ2 subunit gene in parvalbumin-expressing neurons. (A)** Learning the rotarod performance. The rotarod was accelerated from 5 to 20 rpm during 180 s. Data for γ2I77lox control (*n* = 8) and Pv-Δγ2 (*n* = 7) mice are presented as daily means of 6 trials ± SEM. ^#^*P* < 0.05 for the significance of the difference between the mouse lines on training days 1 and 2 (repeated measures ANOVA and Newman–Keuls *post hoc* tests). **(B)** Effects of pregnanolone on motor performance. Pregnanolone was administered in cumulative doses (10+10+10 mg/kg i.p., total 30 mg/kg) to the same animals and the performance tested as in panel **(A)**. One-way ANOVA and Newman–Keuls *post hoc* tests: ^##^*P* < 0.01, ^###^*P* < 0.001 for the significance of the difference between the mouse lines, ^∗^*P* < 0.05, ^∗∗^*P* < 0.01, ^∗∗∗^*P* < 0.001 for the significance of the difference from vehicle injection within the Pv-Δγ2 line. Data are presented as means ± SEM.

A mouse line [Pv-Δγ2, (Wulff et al., 2009a)] with inactivated synaptic GABA_A_ receptor-mediated inhibition of Pv-expressing neurons exhibited a high sensitivity to the exogenous neuroactive steroid 5β-pregnan-3α-ol-20-one (pregnanolone; **Figure 1**). Naive Pv-Δγ2 mice exhibited ataxia, but they could learn to stay on a rotating rod that accelerated from 5 to 20 rpms (**Figure 1A**). However, when these mice were challenged by cumulative dosing of pregnanolone they quickly fell down from the rod, while the control littermates were only slightly affected (**Figure 1B**, drug treatment × genotype interaction *F*_3,53_ = 5.33, *P* < 0.01). Electrophysiology of the Pv-expressing hippocampal interneurons in the genetically engineered mice indicated abolition of synaptic GABA_A_ receptor-mediated inhibitory postsynaptic currents (IPSCs) (Wulff et al., 2009a). Pv-expression is widespread in the brain, including the cerebellar PCs (Meyer et al., 2002; Leppa et al., 2011; Kaiser et al., 2015). Thus, it seemed possible that neurosteroids can induce strong neuronal effects especially in the absence of their target receptors from a set of synapses.

**FIGURE 2:**
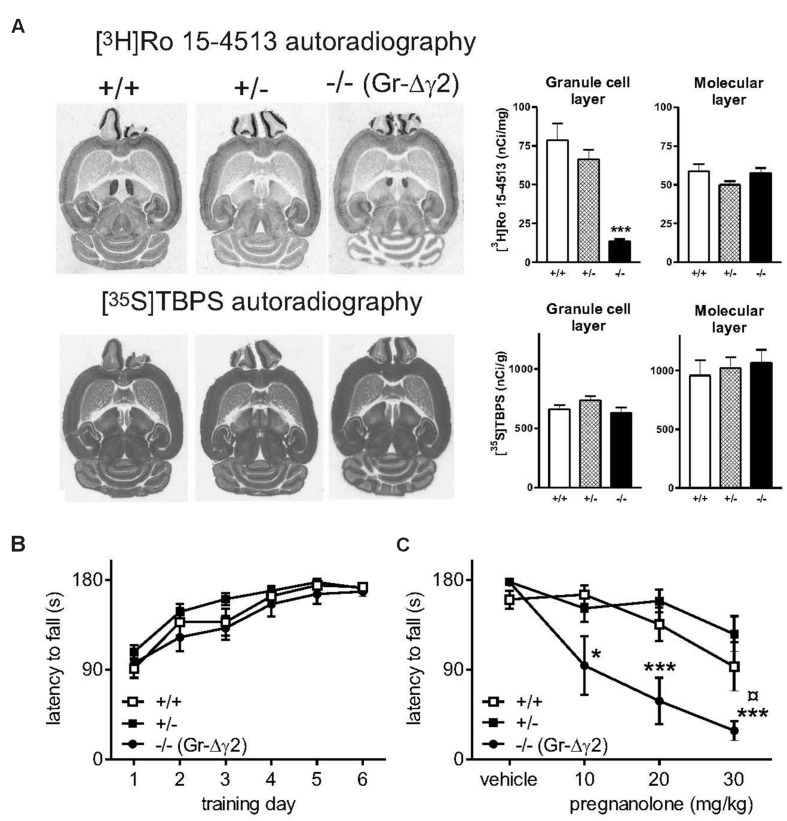
**Neurosteroid sensitivity of Gr-Δγ2 mice with targeted inactivation of GABA_A_ receptor γ2 subunit gene in the cerebellar granule cells. (A)** Ligand binding sites in horizontal brain sections from Gr-Δγ2 mice with the wildtype γ2 subunit background. [^3^H]Ro 15-4513 labels the γ2 subunit-dependent flumazenil-sensitive benzodiazepine sites, which are deficient in the cerebellar granule cell layer of the genetically engineered mice, but not in the molecular layer (as shown in the bar graph). [^35^S]TBPS labels the picrotoxin-sensitive ionophore sites of the GABA_A_ receptor, and this labeling is intact in the cerebellum of the genetically engineered mice, suggesting normal amounts of α and β subunit expression. **(B)** Rotarod learning. The rotarod was accelerated from 5 to 30 rpm during 180 s. Data for wild-type +/+ (*n* = 5), heterozygous +/- (*n* = 10) and Gr-Δγ2 knockout -/- (*n* = 6) mice are presented as daily means of 6 trials ± SEM. There were no significant differences between mouse lines. **(C)** Effects of pregnanolone on rotarod performance. Pregnanolone was administered in cumulative doses (10+10+10 mg/kg i.p., total 30 mg/kg) to the same animals, and the performance tested, as in panel **(B)**. ANOVA followed by Newman–Keuls *post hoc* tests: ^¤^*P* < 0.05 for the significance of the difference of +/+ and +/- mice from the -/- mice. ^∗^*P* < 0.05, ^∗∗∗^*P* < 0.001 for the significance of the difference compared to vehicle within lines. Data are presented as means ± SEM.

Since Pv-expressing neurons exist at a low and diffuse level in most areas of the central nervous system, including motor neurons of the brain stem and spinal cord, and because they have abundant connections between each other and to principal neurons throughout the brain, interpretation of the effect of global Pv-cell-specific elimination of synaptic inhibition is complicated. Thus, we next wanted to use a mouse model with a more restricted ablation of synaptic inhibition. We produced cerebellar granule cell-selective GABA_A_ receptor γ2 subunit knockout mice (Gr-Δγ2) by cross-breeding α6-Cre (Aller et al., 2003) and floxed γ2 mice (Schweizer et al., 2003). Autoradiography of cerebellar sections showed that in the granule cell layer, the Gr-Δγ2 knockouts lack the γ2 subunit-dependent high-affinity labeling of the benzodiazepine binding sites, but contain normal labeling of integral ionophore sites of GABA_A_ receptors (**Figure 2A**). This is consistent with the loss of γ2 subunit-containing receptors at synapses between GABAergic Golgi interneurons and granule cells. In spite of these deficits in synaptic receptors, the Gr-Δγ2 mice learnt normally to run on a rotarod and did not show obvious motor deficits (**Figure 2B**). Interestingly, these mice showed a significant increase in pregnanolone sensitivity as compared to littermate controls and heterozygous knockouts (**Figure 2C**; drug treatment × genotype interaction *F*_6,72_ = 2.87, *P* < 0.05).

**FIGURE 3:**
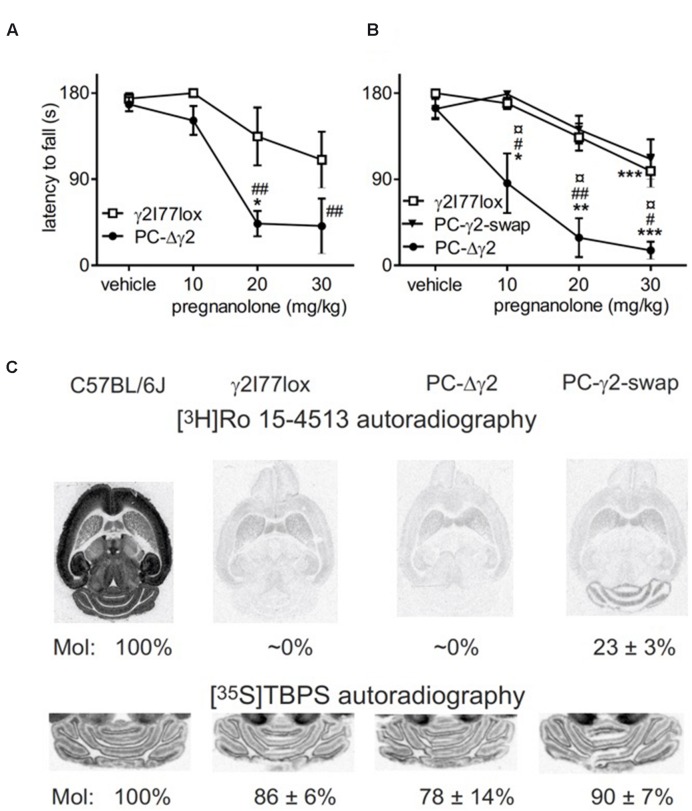
**Neurosteroid sensitivity of PC-Δγ2 mice with targeted inactivation of GABA_A_ receptor γ2 subunit gene in the cerebellar Purkinje neurons. (A)** Effects of pregnanolone on motor performance. Pregnanolone was administered in cumulative doses (10+10+10 mg/kg i.p., total 30 mg/kg) to the control γ2I77lox (*n* = 5) and PC-Δγ2 (*n* = 5) mice, and the performance tested on accelerating rotarod (from 5 to 30 rpm) during 180 s. ANOVA followed by Newman–Keuls *post hoc* tests: ^##^*P* < 0.01 for the significance of the difference between the mouse lines, ^∗^*P* < 0.05 for the significance of the difference compared to vehicle within the PC-Δγ2 line. Data are presented as means ± SEM. **(B)** Effects of wildtype γ2 subunit rescue to the Purkinje neurons in PC-γ2-swap mice on pregnanolone sensitivity. Drug administration and testing of γ2I77lox (*n* = 10), PC-Δγ2 (*n* = 5) and PC-γ2-swap (*n* = 6) mice were carried out as in panel **(A)**. ANOVA followed by Newman–Keuls *post hoc* tests: ^#^*P* < 0.05, ^##^*P* < 0.01 for the significance of the difference between PC-Δγ2 and γ2I77lox mice. ¤*P* < 0.05 for the significance of the difference between PC-Δγ2 and PC-γ2-swap mouse lines. ^∗^*P* < 0.05, ^∗∗^*P* < 0.01, ^∗∗∗^*P* < 0.001 for the significance of the difference compared to vehicle injection within the mouse lines. Data are presented as means ± SEM. **(C)** Representative images of γ2 subunit-dependent [^3^H]Ro 15-4513 binding in normal wildtype C57BL/6J, γ2I77lox, PC-Δγ2 and PC-γ2-swap mouse horizontal brain sections, demonstrating a clear rescue of molecular layer binding in PC-γ2-swap mice. Please, note the very low binding in the forebrain regions in the brains with γ2I77lox background as compared to the wildtype brain. Representative cerebellar images of GABA_A_ receptor ionophore binding sites labeled with [^35^S]TBPS show unchanged binding in genetically engineered mice.

Our third mouse model focused on manipulating inhibitory synaptic input to cerebellar Purkinje cells. We performed motor tests in male PC-Δγ2 mice (Wulff et al., 2007). These mice lack the spontaneous IPSCs of Purkinje neurons (Wulff et al., 2007), but they exhibited only a minor transient motor impairment during the 1st day of rotarod training. The motor-impairing effect of pregnanolone on rotarod performance was clearly enhanced in PC-Δγ2 mice as compared to littermates (**Figures 3A,B**; drug treatment × genotype interaction, *F*_6,69_ = 2.96, *P* = 0.05). Importantly, the re-introduction of wildtype γ2 subunits to PCs, as evidenced by autoradiographic (**Figure 3C**) and electrophysiological experiments (Wulff et al., 2007), fully abolished the increased pregnanolone sensitivity (**Figure 3B**; *P* > 0.05 for the difference between PC-γ2-swap and γ2I77lox lines). These results finally implicate PCs as targets of pregnanolone in the cerebellar cortex resulting in impaired motor coordination.

**FIGURE 4:**
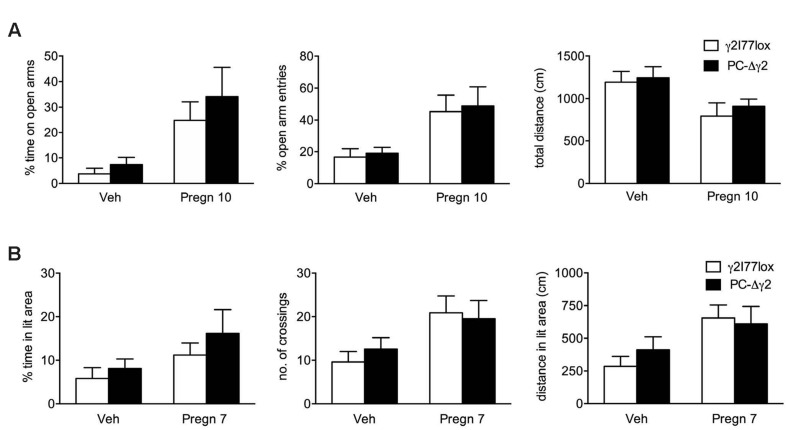
**Effects of pregnanolone on PC-Δγ2 mice with targeted inactivation of GABA_A_ receptor γ2 subunit gene in the cerebellar Purkinje neurons in tests for anxiety and exploration.** The γ2I77lox and PC-Δγ2 mice were treated with vehicle or pregnanolone (at doses indicated below the bars, in mg/kg i.p.) and 15 min later tested in the elevated plus maze **(A)** and light:dark exploration **(B)** tests. The results (means ± SEM, *n* = 7–9 per group) show no differences between mouse lines (two-way ANOVA, *P* > 0.1), but significant effects by the treatment in both tests for all measures (*P* < 0.013), except for the % time in the lit area (*P* = 0.07).

To test whether PC-Δγ2 mice show enhanced sensitivity in other than motor coordination tests, doses of pregnanolone that are not strongly motor impairing (7 and 10 mg/kg) were tested for possible anxiolytic/exploration-increasing effects in the elevated plus maze and light:dark exploration tests, but the results were essentially similar in γ2I77lox and PC-Δγ2 mice (**Figure 4**), thus showing no baseline differences in any measures, nor any significant mouse line or interaction effects in the ANOVA. Pregnanolone treatment induced an anxiolytic-like effect that was similar in both mouse lines. It significantly increased the time on the open arms (*F*_1,29_ = 11.8, *P* = 0.002) and the proportion of open arm entries (*F*_1,29_ = 12.2, *P* = 0.0015) and reduced the distance moved (*F*_1,29_ = 7.6, *P* = 0.01) in the plus maze test, and it tended to increase the time in the lit compartment (*F*_1,31_ = 3.44, *P* = 0.07), increased number of crossings between the compartments (*F*_1,31_ = 7.07, *P* = 0.012) and increased distance traveled in the lit compartment (*F*_1,31_ = 7.34, *P* = 0.010) in the light:dark test. Furthermore, PC-Δγ2 mice showed no reduction in locomotor activity in these tests as compared to controls.

**FIGURE 5:**
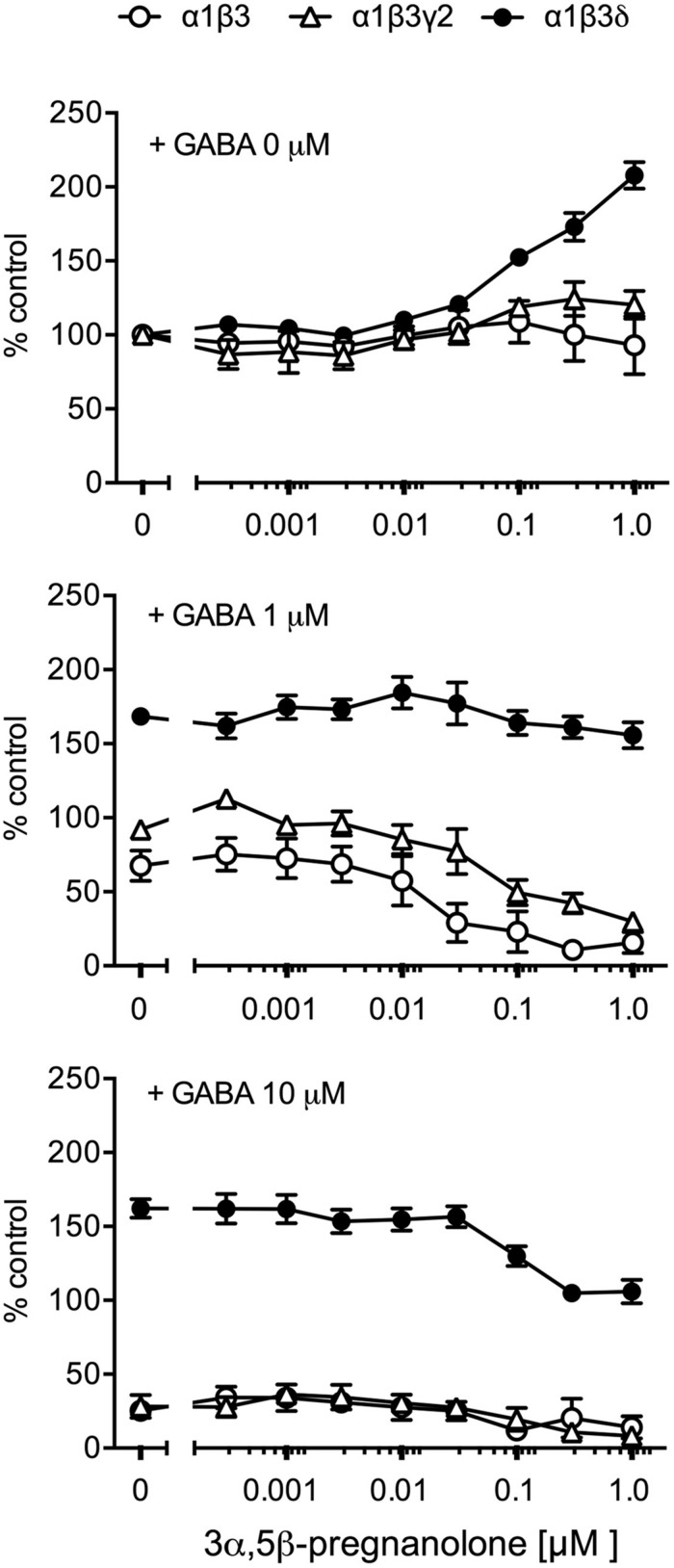
**Effects of pregnanolone on GABA_A_ receptor subtypes in the presence and absence of GABA.** GABA_A_ receptors of the given subunit combinations were transiently expressed in HEK 293 cells and incubated with 3 nM [^3^H]EBOB in the presence and absence of GABA and increasing concentrations of pregnanolone, as indicated. Data are presented as the means ± SEM, *n* = 3–5. All data were normalized to the [^3^H]EBOB binding with setting 0-μM GABA + 0-μM pregnanolone to 100%.

Purkinje cells express especially the α1 subunit-containing GABA_A_ receptors (Wisden et al., 1996). Therefore, we used recombinant GABA_A_ receptors to assess whether different α1 subunit-containing receptor subtypes are affected by the presence of pregnanolone. In the absence of GABA, among α1β3, α1β3γ2, and α1β3δ receptors only the δ subunit-containing receptor α1β3δ responded to increasing concentrations of pregnanolone with increased binding of [^3^H]EBOB (**Figure 5**). At 1 μM GABA, pregnanolone reduced [^3^H]EBOB binding to α1β3 and α1β3γ2 receptors (**Figure 5**), but had no effects on α1β3δ. At the highest GABA concentration tested (10 μM), high nanomolar pregnanolone reduced the binding to α1β3δ receptors but had no more effects on the already low binding to α1β3 and α1β3γ2 receptors. These data suggest that also in a biochemical assay of recombinant GABA_A_ receptor function, the δ subunit-containing receptors respond strongly to the presence of pregnanolone. More importantly, the α1β3 receptors that do not have the address to go to synapses are as strongly affected as α1β3γ2 receptors by pregnanolone.

## Discussion

Neurosteroids are potent modulators of brain GABA_A_ receptors and their significance to various behavioral states is under active investigation. The level of endogenous neurosteroids is thought to influence neuronal excitability, e.g., in catamenial epilepsy, premenstrual syndrome and migraine as well as depression (van Broekhoven and Verkes, 2003; Wu et al., 2013; Backstrom et al., 2014). Neurosteroid mechanisms seem to be important also in liver failure and hepatic encephalopathy (Ahboucha et al., 2012) and in Alzheimer’s disease models (Wang et al., 2010; Bengtsson et al., 2012, 2013; Zhang et al., 2015). Using several GABA_A_ receptor genetically engineered mouse models we show here that the neuroactive steroid agonist pregnanolone can strongly modulate learned motor performance when the balance of non-synaptic (extrasynaptic) and synaptic GABA_A_ receptors was altered in a set of neuronal populations with inactivated γ2 subunits. Previous findings on α4 and δ subunit-containing receptors in hippocampal areas indicate that neurosteroid agonists powerfully facilitate tonic inhibition mediated by GABA_A_ receptors (Carver et al., 2014). These results, together with the reduced sensitivity of neurosteroids in GABA_A_ receptor δ knockout mice (Mihalek et al., 1999; Stell et al., 2003), strongly indicate that the extrasynaptic GABA_A_ receptors are the preferential targets of neurosteroids. Importantly, in the present study these preferential effects could be detected in mice with impaired synaptic inhibition only in a single population of neurons after inactivation of the γ2 subunit that is essential for synaptic targeting of GABA_A_ receptors (Crestani et al., 1999), without any general enhancement of δ subunit-containing GABA_A_ receptors. It should anyway be kept in mind that compensatory changes may take place after inactivation of γ2 subunits, although the δ subunit is not known to be expressed in the cerebellar PCs (Wisden et al., 1996) and global γ2 inactivation cannot be efficiently compensated in mice (Gunther et al., 1995).

The modulation of binding of the GABA_A_ receptor ionophore ligand [^3^H]EBOB was different in the δ subunit-containing receptors than in binary αβ or γ2 subunit-containing receptors (**Figure 5**), again indicating a possibility that δ subunit-containing GABA_A_ receptors have features that make neurosteroid effects in them more potent or efficient. Further study is needed to understand the molecular mechanisms how the general neurosteroid binding sites on α subunits (Hosie et al., 2009) are transducing the signal to the ionophore structure in the δ subunit-containing receptors differently from δ subunit-absent receptors.

The alteration of balance between synaptic and extrasynaptic inhibition might be also operative in the regulation of other neuronal pathways that mediate more subtle behaviors such as emotions and irritability, that are known to be associated with altered endogenous neurosteroid metabolism and GABAergic inhibition (Backstrom et al., 2003). In the present study the anxiolytic and exploration-related effects of pregnanolone were not affected by the γ2 inactivation in the cerebellar PCs (**Figure 4**). The tests should be extended in further studies by using more appropriate neuronal populations for emotional regulation, e.g., in the forebrain.

Learned motor performance depends on cerebellar circuitry. In a recent study, the conditioned eyeblink reflex was pinpointed to deep cerebellar nuclei, particularly the anterior interpositus nuclei, which receive input from cerebellar cortical PCs (Ito, 1984; Heiney et al., 2014). PCs have multiple roles in movement precision, including predictive activity and instructive signaling downstream to the deep cerebellar nuclei. Optogenetic activation of PCs-inhibiting interneurons in the molecular layer transiently decreases the constitutive inhibitory output of PCs to the deep cerebellar nuclei and consequently produces precisely timed movement activation in awake mice (Heiney et al., 2014). Our present findings of enhanced motor impairment by pregnanolone acting on non-synaptic GABA_A_ receptors in various cerebellar cortical neuron populations likely resulted from non-timed prolonged disinhibition of the deep cerebellar nuclei.

In Pv-Δγ2 mice the predicted absence of synaptic inhibition with maintained extrasynaptic inhibition in widespread brain regions resulted in a pronounced increase in pregnanolone sensitivity in the rotarod test. Importantly, cerebellar molecular layer interneurons, such as stellate and basket cells are Pv-positive, as are the PCs as well (Meyer et al., 2002; Kaiser et al., 2015), and the inhibitory feed-forward control the molecular layer interneurons exert on PCs via synaptic GABA_A_ receptors is perforce altered in Pv-Δγ2 mice (Wulff et al., 2009b). When pregnanolone is administered, it affects the cerebellar function in these knockout mice more strongly than in wild-type mice. Considering that the synaptic inhibitory influence of molecular layer interneurons on PCs is already lacking, the effect of pregnanolone on remaining extrasynaptic receptors in both PCs and stellate/basket cells produces a network inhibitory effect that impairs the learned motor behavior of Pv-Δγ2 mice.

Pregnanolone produced a robustly increased effect on learned motor performance also in Gr-Δγ2 mice. Despite the missing synaptic inhibition from Golgi interneurons to granule cells (correlating with the absence of α1/6βγ2 subunit-dependent [^3^H]Ro 15-4513 binding, **Figure 2A**), these mice learned motor tasks as well as wild-type mice, suggesting compensations. Normally cerebellar granule cells have an excitatory effect on Purkinje cells via parallel fiber axons. When Gr-Δγ2 mice are administered pregnanolone, this excitation is possibly reduced to a greater extent and/or the phasic changes in PC firing are blunted compared to wild-type mice due to the prevalence of only extrasynaptic receptors in the granule cells. The resulting decrease in timed PC activity is a likely explanation for the increased impairing effect of pregnanolone on motor performance. This cerebellar granule-cell specific mouse model revealed that ablation of phasic inhibition only in a single population of cerebellar neurons, although in the most abundant one of the brain, is enough to bring about enhanced neurosteroid sensitivity.

Purkinje cell-Δγ2 mice were previously found to have disrupted inhibitory and excitatory input timing from PCs and mossy fiber collaterals (Wulff et al., 2009b). The lack of the GABA_A_ γ2 subunit disrupts the coordination of PC activity through molecular layer interneurons. In the present study powerfully prolonged tonic inhibition of PC activity by pregnanolone acting on extrasynaptic GABA_A_ receptors would disrupt the learning and performance of motor tasks more in PC-Δγ2 than in wild-type mice due to the sole availability of presumably α1β2 extrasynaptic receptors. Compensatory mechanisms, such as up-regulation of δ subunit expression, cannot be excluded, though. In PC-γ2-swap mice the situation was normalized due to the restoration of synaptic GABAergic inhibition in the molecular layer. As compared to the normal γ2F77 wild-type mice, the rescue of γ2-dependent benzodiazepine site binding in PC-γ2-swap was only 23% (**Figure 3C**), but this comparison may underestimate the rescue, since it does not take into account the binding to molecular layer interneurons missing at the γ2I77 background. Anyway, this mouse model with the rescue experiment pinpointed that the presence of γ2 subunits and associated synaptic inhibition strongly blunted the effect of pregnanolone on motor performance, underscoring the importance of phasic, directly neurotransmission-linked inhibition. Each PC receives a robust, convergent inhibition from about seven GABAergic, gap-junctionally connected interneurons in the molecular layer (Hausser and Clark, 1997; Park et al., 2012; Kim et al., 2014). The abolition of this phasic function should at least affect the timing of inhibition and thereby the regulation of firing of different PCs and at the end the motor performance. In the absence of γ2 subunits, pregnanolone presumably induced prolonged inhibition of PC firing via extrasynaptic receptors. Alternative mechanisms could involve cerebellar glutamatergic N-methyl-D-aspartate receptors that might undergo adaptive changes in neurons with deficient synaptic inhibition [synaptic scaling; cf. (Moykkynen et al., 2007)], especially as some neurosteroids blunt cerebellar responses to glutamate via potentiating GABA_A_ receptor functions or by blocking glutamate receptors (Cauli et al., 2011; Bali and Jaggi, 2014; Vyklicky et al., 2015).

## Conclusion

Taken as a whole, these results from four different genetically engineered mouse lines with disruptions in the synaptic connections of the cerebellar network point to a delicate balance of inhibitory and excitatory input toward PCs. A possible interpretation of our results is that reduced inhibition from PCs toward the deep cerebellar nuclei, resulting from either decreased excitation (Gr-Δγ2 mice) or increased sustained inhibition of PCs (Pv-Δγ2 and PC-Δγ2 mice), caused the increased effect of pregnanolone on the rotarod test performance. These mouse models may form the basis for studies on molecular mechanisms of neurosteroid actions and for detailed understanding of how the cerebellar cortex regulates motor performance.

## Author Contributions

EL, A-ML, PW, BL, WW, and EK designed the experiments on mouse models and autoradiography. HL designed and carried out the recombinant receptor study. EL, A-ML, MA, and OV carried out the experiments. All authors wrote and approved the manuscript.

## Conflict of Interest Statement

The authors declare that the research was conducted in the absence of any commercial or financial relationships that could be construed as a potential conflict of interest.

## References

[B1] Abramian A. M. Comenencia-Ortiz E. Modgil A. Vien T. N. Nakamura Y. Moore Y. E. (2014). Neurosteroids promote phosphorylation and membrane insertion of extrasynaptic GABA_A_ receptors. *Proc. Natl. Acad. Sci. U.S.A.* 111 7132–7137. 10.1073/pnas.1403285111 24778259 PMC4024867

[B2] Ahboucha S. Gamrani H. Baker G. (2012). GABAergic neurosteroids: the “endogenous benzodiazepines” of acute liver failure. *Neurochem. Int.* 60 707–714. 10.1016/j.neuint.2011.10.003 22041164

[B3] Aller M. I. Jones A. Merlo D. Paterlini M. Meyer A. H. Amtmann U. (2003). Cerebellar granule cell Cre recombinase expression. *Genesis* 36 97–103. 10.1002/gene.10204 12820171

[B4] Backstrom T. Andersson A. Andree L. Birzniece V. Bixo M. Bjorn I. (2003). Pathogenesis in menstrual cycle-linked CNS disorders. *Ann. N. Y. Acad. Sci.* 1007 42–53. 10.1196/annals.1286.005 14993039

[B5] Backstrom T. Bixo M. Johansson M. Nyberg S. Ossewaarde L. Ragagnin G. (2014). Allopregnanolone and mood disorders. *Prog. Neurobiol.* 113 88–94. 10.1016/j.pneurobio.2013.07.005 23978486

[B6] Backstrom T. Haage D. Lofgren M. Johansson I. M. Stromberg J. Nyberg S. (2011). Paradoxical effects of GABA-A modulators may explain sex steroid induced negative mood symptoms in some persons. *Neuroscience* 191 46–54. 10.1016/j.neuroscience.2011.03.061 21600269

[B7] Bali A. Jaggi A. S. (2014). Multifunctional aspects of allopregnanolone in stress and related disorders. *Prog. Neuropsychopharmacol. Biol. Psychiatry* 48 64–78. 10.1016/j.pnpbp.2013.09.005 24044974

[B8] Barbaccia M. L. (2004). Neurosteroidogenesis: relevance to neurosteroid actions in brain and modulation by psychotropic drugs. *Crit. Rev. Neurobiol.* 16 67–74. 10.1615/CritRevNeurobiol.v16.i12.70 15581401

[B9] Barski J. J. Dethleffsen K. Meyer M. (2000). Cre recombinase expression in cerebellar Purkinje cells. *Genesis* 28 93–98. 10.1002/1526-968X(200011/12)28:3/4<93::AID-GENE10>3.3.CO;2-N 11105049

[B10] Baulieu E. E. Robel P. Schumacher M. (2001). Neurosteroids: beginning of the story. *Int. Rev. Neurobiol.* 46 1–32. 10.1016/S0074-7742(01)46057-0 11599297

[B11] Belelli D. Casula A. Ling A. Lambert J. J. (2002). The influence of subunit composition on the interaction of neurosteroids with GABA(A) receptors. *Neuropharmacology* 43 651–661. 10.1016/S0028-3908(02)00172-7 12367610

[B12] Belelli D. Herd M. B. Mitchell E. A. Peden D. R. Vardy A. W. Gentet L. (2006). Neuroactive steroids and inhibitory neurotransmission: mechanisms of action and physiological relevance. *Neuroscience* 138 821–829. 10.1016/j.neuroscience.2005.07.021 16310966

[B13] Bengtsson S. K. Johansson M. Backstrom T. Nitsch R. M. Wang M. (2013). Brief but chronic increase in allopregnanolone cause accelerated AD pathology differently in two mouse models. *Curr. Alzheimer Res.* 10 38–47. 10.2174/156720513804871363 23157375

[B14] Bengtsson S. K. Johansson M. Backstrom T. Wang M. (2012). Chronic allopregnanolone treatment accelerates Alzheimer’s disease development in AbetaPP(Swe)PSEN1(DeltaE9) mice. *J. Alzheimers. Dis.* 31 71–84. 22495347 10.3233/JAD-2012-120268

[B15] Carver C. M. Wu X. Gangisetty O. Reddy D. S. (2014). Perimenstrual-like hormonal regulation of extrasynaptic delta-containing GABA_A_ receptors mediating tonic inhibition and neurosteroid sensitivity. *J. Neurosci.* 34 14181–14197. 10.1523/JNEUROSCI.0596-14.2014 25339733 PMC4205546

[B16] Cauli O. Gonzalez-Usano A. Agusti A. Felipo V. (2011). Differential modulation of the glutamate-nitric oxide-cyclic GMP pathway by distinct neurosteroids in cerebellum in vivo. *Neuroscience* 190 27–36. 10.1016/j.neuroscience.2011.06.009 21703332

[B17] Cope D. W. Halbsguth C. Karayannis T. Wulff P. Ferraguti F. Hoeger H. (2005). Loss of zolpidem efficacy in the hippocampus of mice with the GABA_A_ receptor gamma2 F77I point mutation. *Eur. J. Neurosci.* 21 3002–3016. 10.1111/j.1460-9568.2005.04127.x 15978011

[B18] Cope D. W. Wulff P. Oberto A. Aller M. I. Capogna M. Ferraguti F. (2004). Abolition of zolpidem sensitivity in mice with a point mutation in the GABA_A_ receptor gamma2 subunit. *Neuropharmacology* 47 17–34. 10.1016/j.neuropharm.2004.03.007 15165831

[B19] Crestani F. Lorez M. Baer K. Essrich C. Benke D. Laurent J. P. (1999). Decreased GABA_A_-receptor clustering results in enhanced anxiety and a bias for threat cues. *Nat. Neurosci.* 2 833–839. 10.1038/12207 10461223

[B20] Fuchs E. C. Zivkovic A. R. Cunningham M. O. Middleton S. Lebeau F. E. Bannerman D. M. (2007). Recruitment of parvalbumin-positive interneurons determines hippocampal function and associated behavior. *Neuron* 53 591–604. 10.1016/j.neuron.2007.01.031 17296559

[B21] Gunther U. Benson J. Benke D. Fritschy J. M. Reyes G. Knoflach F. (1995). Benzodiazepine-insensitive mice generated by targeted disruption of the gamma 2 subunit gene of gamma-aminobutyric acid type a receptors. *Proc. Natl. Acad. Sci. U.S.A.* 92 7749–7753. 10.1073/pnas.92.17.7749 7644489 PMC41223

[B22] Hausser M. Clark B. A. (1997). Tonic synaptic inhibition modulates neuronal output pattern and spatiotemporal synaptic integration. *Neuron* 19 665–678. 10.1016/S0896-6273(00)80379-7 9331356

[B23] Heiney S. A. Kim J. Augustine G. J. Medina J. F. (2014). Precise control of movement kinematics by optogenetic inhibition of Purkinje cell activity. *J. Neurosci.* 34 2321–2330. 10.1523/JNEUROSCI.4547-13.2014 24501371 PMC3913874

[B24] Hosie A. M. Clarke L. da Silva H. Smart T. G. (2009). Conserved site for neurosteroid modulation of GABA a receptors. *Neuropharmacology* 56 149–154. 10.1016/j.neuropharm.2008.07.050 18762201

[B25] Hosie A. M. Wilkins M. E. da Silva H. M. Smart T. G. (2006). Endogenous neurosteroids regulate GABA_A_ receptors through two discrete transmembrane sites. *Nature* 444 486–489. 10.1038/nature05324 17108970

[B26] Ito M. (1984). *The Cerebellum and Neural Control.* New York, NY: Raven Press.

[B27] Jordan M. Schallhorn A. Wurm F. M. (1996). Transfecting mammalian cells: optimization of critical parameters affecting calcium-phosphate precipitate formation. *Nucleic Acids Res.* 24 596–601. 10.1093/nar/24.4.596 8604299 PMC145683

[B28] Kaiser T. Ting J. T. Monteiro P. Feng G. (2015). Transgenic labeling of parvalbumin-expressing neurons with tdTomato. *Neuroscience* 21 236–245. 10.1016/j.neuroscience.2015.08.036 PMC4769998 26318335

[B29] Kim J. Lee S. Tsuda S. Zhang X. Asrican B. Gloss B. (2014). Optogenetic mapping of cerebellar inhibitory circuitry reveals spatially biased coordination of interneurons via electrical synapses. *Cell Rep.* 7 1601–1613. 10.1016/j.celrep.2014.04.047 24857665 PMC4107211

[B30] Korpi E. R. Koikkalainen P. Vekovischeva O. Y. Makela R. Kleinz R. Uusi-Oukari M. (1999). Cerebellar granule-cell-specific GABA_A_ receptors attenuate benzodiazepine-induced ataxia: evidence from alpha 6-subunit-deficient mice. *Eur. J. Neurosci.* 11 233–240. 10.1046/j.1460-9568.1999.00421.x 9987027

[B31] Korpi E. R. Luddens H. (1997). Furosemide interactions with brain GABA_A_ receptors. *Br. J. Pharmacol.* 120 741–748. 10.1038/sj.bjp.0700922 9138676 PMC1564522

[B32] Leppa E. Linden A. M. Vekovischeva O. Y. Swinny J. D. Rantanen V. Toppila E. (2011). Removal of GABA(A) receptor gamma2 subunits from parvalbumin neurons causes wide-ranging behavioral alterations. *PLoS ONE* 6:e24159 10.1371/journal.pone.0024159 PMC3166293 21912668

[B33] Leppa E. Vekovischeva O. Y. Linden A. M. Wulff P. Oberto A. Wisden W. (2005). Agonistic effects of the beta-carboline DMCM revealed in GABA(A) receptor gamma 2 subunit F77I point-mutated mice. *Neuropharmacology* 48 469–478. 10.1016/j.neuropharm.2004.11.007 15755475

[B34] Linden A. M. Schmitt U. Leppa E. Wulff P. Wisden W. Luddens H. (2011). Ro 15-4513 antagonizes alcohol-induced sedation in mice through alphabetagamma2-type GABA(A) receptors. *Front. Neurosci.* 5:3 10.3389/fnins.2011.00003 PMC3026482 21270945

[B35] Mackenzie G. Maguire J. (2014). The role of ovarian hormone-derived neurosteroids on the regulation of GABA receptors in affective disorders. *Psychopharmacology (Berl.)* 231 3333–3342. 10.1007/s00213-013-3423-z 24402140 PMC4090295

[B36] Maguire J. L. Stell B. M. Rafizadeh M. Mody I. (2005). Ovarian cycle-linked changes in GABA(A) receptors mediating tonic inhibition alter seizure susceptibility and anxiety. *Nat. Neurosci.* 8 797–804. 10.1038/nn1469 15895085

[B37] Makela R. Uusi-Oukari M. Homanics G. E. Quinlan J. J. Firestone L. L. Wisden W. (1997). Cerebellar gamma-aminobutyric acid type a receptors: pharmacological subtypes revealed by mutant mouse lines. *Mol. Pharmacol.* 52 380–388. 9281599 10.1124/mol.52.3.380

[B38] Meyer A. H. Katona I. Blatow M. Rozov A. Monyer H. (2002). In vivo labeling of parvalbumin-positive interneurons and analysis of electrical coupling in identified neurons. *J. Neurosci.* 22 7055–7064. 12177202 10.1523/JNEUROSCI.22-16-07055.2002 PMC6757887

[B39] Mihalek R. M. Banerjee P. K. Korpi E. R. Quinlan J. J. Firestone L. L. Mi Z. P. (1999). Attenuated sensitivity to neuroactive steroids in gamma-aminobutyrate type A receptor delta subunit knockout mice. *Proc. Natl. Acad. Sci. U.S.A.* 96 12905–12910. 10.1073/pnas.96.22.12905 10536021 PMC23157

[B40] Moykkynen T. P. Sinkkonen S. T. Korpi E. R. (2007). Compensation by reduced L-alpha-amino-3-hydroxy-5-methyl-4-isoxazolepropionic acid receptor responses in a mouse model with reduced gamma-aminobutyric acid type a receptor-mediated synaptic inhibition. *J. Neurosci. Res.* 85 668–672. 10.1002/jnr.21138 17131399

[B41] Park S. M. Tara E. Khodakhah K. (2012). Efficient generation of reciprocal signals by inhibition. *J. Neurophysiol.* 107 2453–2462. 10.1152/jn.00083.2012 22298833 PMC3362251

[B42] Pritchett D. B. Seeburg P. H. (1990). Gamma-aminobutyric acidA receptor alpha 5-subunit creates novel type II benzodiazepine receptor pharmacology. *J. Neurochem.* 54 1802–1804. 10.1111/j.1471-4159.1990.tb01237.x 2157817

[B43] Saarelainen K. S. Ranna M. Rabe H. Sinkkonen S. T. Moykkynen T. Uusi-Oukari M. (2008). Enhanced behavioral sensitivity to the competitive GABA agonist, gaboxadol, in transgenic mice over-expressing hippocampal extrasynaptic alphα6beta GABA(A) receptors. *J. Neurochem.* 105 338–350. 10.1111/j.1471-4159.2007.05136.x 18021290

[B44] Schweizer C. Balsiger S. Bluethmann H. Mansuy I. M. Fritschy J. M. Mohler H. (2003). The gamma 2 subunit of GABA(A) receptors is required for maintenance of receptors at mature synapses. *Mol. Cell. Neurosci.* 24 442–450. 10.1016/S1044-7431(03)00202-1 14572465

[B45] Sieghart W. (1995). Structure and pharmacology of gamma-aminobutyric acidA receptor subtypes. *Pharmacol. Rev.* 47 181–234. 7568326

[B46] Spigelman I. Li Z. Liang J. Cagetti E. Samzadeh S. Mihalek R. M. (2003). Reduced inhibition and sensitivity to neurosteroids in hippocampus of mice lacking the GABA(A) receptor delta subunit. *J. Neurophysiol.* 90 903–910. 10.1152/jn.01022.2002 12702713

[B47] Stell B. M. Brickley S. G. Tang C. Y. Farrant M. Mody I. (2003). Neuroactive steroids reduce neuronal excitability by selectively enhancing tonic inhibition mediated by delta subunit-containing GABA_A_ receptors. *Proc. Natl. Acad. Sci. U.S.A.* 100 14439–14444. 10.1073/pnas.2435457100 14623958 PMC283610

[B48] Uusi-Oukari M. Korpi E. R. (2010). Regulation of GABA(A) receptor subunit expression by pharmacological agents. *Pharmacol. Rev.* 62 97–135. 10.1124/pr.109.002063 20123953

[B49] van Broekhoven F. Verkes R. J. (2003). Neurosteroids in depression: a review. *Psychopharmacology (Berl.)* 165 97–110. 12420152 10.1007/s00213-002-1257-1

[B50] Vyklicky V. Krausova B. Cerny J. Balik A. Zapotocky M. Novotny M. (2015). Block of NMDA receptor channels by endogenous neurosteroids: implications for the agonist induced conformational states of the channel vestibule. *Sci. Rep.* 5:10935 10.1038/srep10935 PMC4471902 26086919

[B51] Wang J. M. Singh C. Liu L. Irwin R. W. Chen S. Chung E. J. (2010). Allopregnanolone reverses neurogenic and cognitive deficits in mouse model of Alzheimer’s disease. *Proc. Natl. Acad. Sci. U.S.A.* 107 6498–6503. 10.1073/pnas.1001422107 20231471 PMC2851948

[B52] Wisden W. Korpi E. R. Bahn S. (1996). The cerebellum: a model system for studying GABA_A_ receptor diversity. *Neuropharmacology* 35 1139–1160. 10.1016/S0028-3908(96)00076-7 9014130

[B53] Wisden W. Murray A. J. McClure C. Wulff P. (2009). Studying cerebellar circuits by remote control of selected neuronal types with GABA(A) receptors. *Front. Mol. Neurosci.* 2:29 10.3389/neuro.02.029.2009 PMC2805427 20076763

[B54] Wohlfarth K. M. Bianchi M. T. Macdonald R. L. (2002). Enhanced neurosteroid potentiation of ternary GABA(A) receptors containing the delta subunit. *J. Neurosci.* 22 1541–1549. 11880484 10.1523/JNEUROSCI.22-05-01541.2002 PMC6758857

[B55] Wu X. Gangisetty O. Carver C. M. Reddy D. S. (2013). Estrous cycle regulation of extrasynaptic delta-containing GABA(A) receptor-mediated tonic inhibition and limbic epileptogenesis. *J. Pharmacol. Exp. Ther.* 346 146–160. 10.1124/jpet.113.203653 23667248 PMC3684839

[B56] Wulff P. Goetz T. Leppa E. Linden A. M. Renzi M. Swinny J. D. (2007). From synapse to behavior: rapid modulation of defined neuronal types with engineered GABA_A_ receptors. *Nat. Neurosci.* 10 923–929. 10.1038/nn1927 17572671 PMC2092503

[B57] Wulff P. Ponomarenko A. A. Bartos M. Korotkova T. M. Fuchs E. C. Bahner F. (2009a). Hippocampal theta rhythm and its coupling with gamma oscillations require fast inhibition onto parvalbumin-positive interneurons. *Proc. Natl. Acad. Sci. U.S.A.* 106 3561–3566. 10.1073/pnas.0813176106 19204281 PMC2637907

[B58] Wulff P. Schonewille M. Renzi M. Viltono L. Sassoe-Pognetto M. Badura A. (2009b). Synaptic inhibition of Purkinje cells mediates consolidation of vestibulo-cerebellar motor learning. *Nat. Neurosci.* 12 1042–1049. 10.1038/nn.2348 19578381 PMC2718327

[B59] Zhang P. Xie M. Q. Ding Y. Q. Liao M. Qi S. S. Chen S. X. (2015). Allopregnanolone enhances the neurogenesis of midbrain dopaminergic neurons in APPswe/PSEN1 mice. *Neuroscience* 290 214–226. 10.1016/j.neuroscience.2015.01.019 25637494

